# The opinions of community-centered engagement and health care during and after COVID-19 pandemic

**DOI:** 10.3389/fpsyt.2023.1168860

**Published:** 2023-05-05

**Authors:** Li-Jie Du

**Affiliations:** Department of Sociology, College of Philosophy and Law and Politics, Shanghai Normal University, Shanghai, China

**Keywords:** community-centered, health care, social determinants of health, COVID-19 pandemic, risk communication and community engagement, psychiatry and psychology, medical sociology

## 1. Introduction

The COVID-19 pandemic still remains an impending and grave threat to the global public health. A number of prominent social determinants in common, including more chronic disease, inefficient health care, shortage of education, and severe overcrowding, have been found to be associated with extremely high COVID-19 cases (1). In the context of attempts to curb the spread of the virus, a great deal of focus has been placed on community mitigation efforts (2).

The COVID-19 pandemic has added another layer of trauma to low-income class communities, who often experience the trauma of a historical legacy of racism that still has not been fully healed in some countries (3). Geographical inaccessibility and socioeconomic inequalities have caused unequal health care use across both urban and rural communities even in the same country and region. Following the experiences of Pakistan, Ethiopia, Brazil, and other countries, a coordinated community workforce can provide effective health and social care support on a large scale (4, 5). The UK has also proposed a large-scale emergency program to train Community Health Workers (CHWs) to provide a long-term model of care (6). Community-centered engagement and health care services play a key role in trying to combat this problem. In addition to professional or trained CHWs, other staffs who were in Community-Based Organizations (CBO), Community Health Centers (CHCs), provided essential health care services and contributed empirical experience to the existing research during and after COVID-19 pandemic.

This article introduces a themed issue focused on COVID-19 pandemic as it relates to community-centered engagement and health care services: (1) Risk communication and community engagement plan; (2) Advancing community-based testing and vaccination programs. It provides the general commentary on them.

## 2. Subsections relevant for the subject

### 2.1. Risk communication and community engagement plan

Community engagement is based on the premise that the voice of the community should be heard as it is empowered to play a meaningful role in the process by which it is affected and the solutions to the community's own problems. It is an essential component of humanitarian assistance, civil society and international development practice (7). Risk communication and community engagement (RCCE) are essential components of a broader health emergency preparedness and response action plan (8). In the context of the COVID-19 pandemic, it encompasses two distinct but interrelated approaches to supporting communities to adopt disease-safe behaviors and to take community action to support ending disease transmission. It includes effective dissemination of scientific information, and also the range of communication actions required through the preparedness, response, and recovery phases, to encourage positive behavior change, and the maintenance of trust (9).

In Singapore, migrant workers who were not covered by the universal health care system are one portion of the vulnerable population. They mostly live in large, diverse, high-density housing, and are not governed by local labor laws regarding minimum wages, employment mobility, and occupational rights (10). RCCE's activities in their community lack coordination and are often led by government authorities and non-profit organizations. Through sustained efforts, the RCCE system has evolved from a grassroots approach to a scientifically effective strategy that is coordinated with national actions and disseminated to large, diverse migrant worker communities (11).

In response to the COVID-19 outbreaks, mostas countries or regions have restricted entry and exit, or imposed blockades in some cities at the beginning of 2020. Even city lockdowns are effective as a short-term tool to contain and slow the pandemic spreading, an important challenge for local governments is to ensure that basic supplies are provided asto the residents of the communities, especially to vulnerable groups. The practical experience in Shanghai of China presented its essential feature duringas urban lockdowns: the community plays an important role in providing basic supplies as the main body of grassroots governance. In complianceas with the government's advocacy of community closure, residents staying in their homes and maintaining social distance, the neighborhood committee hasas recruited many volunteers from residents to carry household goods from the community gate to residents' homes (12). In order to reduce theas number of people gathered, volunteers from each building took out supplies from the temporary storage area and delivered them door-to-door (11). The basic livelihood security program for vulnerable groups was implemented by community-based units. Community engagement is essential for creating aslocal and context-specific community-centered interventions (13); at the same time, community engagement helps to build interpersonal astrusting and fosters interaction and networking among neighbors, which can help protect people's mental health and reduce the risk of isolation, asdepression, and even suicide that come with the closure during a lockdown (14).

### 2.2. Advancing community-based testing and vaccination programs

During the pandemic, Community Health Centers (CHCs) functioned as an important source of health care for low-income and non-privately insured populations, serving as a trusted source of care to engage the communities they served in COVID-19 testing. There have been some successes with testing and vaccination programs in those low-income communities. CHCs have the infrastructure to maintain public health, and their place in the community also means they are a powerful force for health equity, social justice, community pride, and resilience. To meet community needs, the government started testing services in CHCs as the first place. In U.S.A, 97% of CHCs had implemented testing services before October 2020 (15). In one case study which aligns with principles of community-engaged research, it describes a community-partnered strategy to accelerate COVID-19 testing in historically marginalized populations that provides ongoing resources to CHCs for addressing the needs of testing in their communities (16). Following the acceleration of the testing strategies, CHC-community partnerships implemented outreach strategies to support testing in populations at increased risk for COVID-19 (17).

Federally Qualified Health Centers (FQHCs) now comprise the largest primary care network in the United States, that are non-profit, community-directed health care providers serving low-income and medically-underserved communities, many of which provide limited access to psychiatric services currently (18). AltaMed Health Services, one of the largest FQHCS, implemented the COVID-19 vaccine outreach and education initiatives which applied Freirean liberation principles to an integrated model of crisis recovery and community resilience-building (19). Hispanic patients and Non-Hispanic Black have higher risk for COVID-19 infection and hospitalization (20), but have lower rates of COVID-19 vaccination (21). Two simultaneous interventions were conducted at the vaccination site in a racially and ethnically diverse neighborhood in northern Manhattan to address this issue: (1) Reschedule patients through the direct education and outreach service in a CBO. (2) A digital redesign to restrict online self-scheduled vaccinations to locally underserved racial and ethnic patient zip codes (17). The results suggest that the appropriate digital workflow designing for vaccination, may reduce health disparities directly, and such efforts highlight the importance of public health campaigns which was community-based engagement.

During the COVID-19 pandemic, people were asked to take such actions as wearing masks, testing, and vaccination. While these actions were beneficial to individuals in combination with others through community immunization, it cannot be assumed that people were enthusiastic about taking these beneficial actions. The community characteristics that are associated with higher testing rates in a voluntary mass testing scheme implemented in the Italian region of South Tyrol between November 18th and 25th of 2020, shows the key community determinants and characteristics that are associated with higher testing rates, such as socioeconomic status, the convenience, religiosity and social capital (22). In the vaccination campaign, different regions of China have different rates of vaccination and different factors that influence people to vaccinate, which may be due to some complex sociodemographic characteristics. Incentives similar to the testing could be used for vaccination, but may prove to be a challenge, so it is of considerable interest to study fully voluntary participation in vaccination. Vaccination strategies need to be tailored to the gender of the community population, the dissemination of vaccination information to achieve higher levels of COVID-19 vaccination (23).

## 3. Discussion and conclusion

The COVID-19 pandemic has exposed structural social inequalities and systemic inequities in our health care systems (24). There were many inequalities in social determinants and exposure to risk, access to health care, and ability to engage in COVID-19 prevention behaviors (25). The early detection of vulnerable categories, at risk to become ill and develop long-term health status, would help to prevent impacts on overall wellbeing by allocating resources for targeted interventions to manage psychosocial stress and increase the resilience of vulnerable populations toward post-COVID-19 crises (26). Public health agencies and health care providers should consider strengths, challenges, the needs of specific communities, and avoid using a uniform “one size fits all” approach when tackling all issues related to COVID-19 (2).

Up to now, the COVID-19 pandemic has greatly imposed stressful conditions that may affect the ability of community health care providers to provide safe and effective care (27). It challenged the community-centered health care providers and inspired new ideas. The concept of resilience which is widely used in various academic fields could also be implemented in the community. The experience of community engagement of the migrant workers in Singapore and the volunteers from residents in Shanghai of China, which were described in the above, have also shown that coordinated and timely RCCE in response could be achieved by establishing specific systems and structures, even in crisis settings where the concept of RCCE is not understood (11).

The findings of the community-centered intervention in testing and vaccination programs showed some significant changes in the racial and ethnic composition of COVID-19 before and after these interventions. Community Health Workers (CHWs), whose close relationship with community members assists in bridging the gap between the community and the health care system, have been shown to play a critical role in limiting the spread of the virus during the pandemic (5). There were other staffs who were in CBO, CHCs, etc. They provided community patient-centered care in the COVID-19 pandemic which could serve as a new starting point for improving and expanding their role in the health care system (28). The COVID-19 provides a window of opportunity for observing community resilience initiatives. The qualitative study, based on the Community Resilience Initiative Framework, investigated the initiatives of urban communities in China (29). The collective experience in fighting the COVID-19 boosted community interaction, understanding and trust. It thus established community self-organization including the agency of community actors, grid management systems, and the utilization of WeChat groups, and further promoted the capacity of problem solving in the community. We will continually apply the concept of resilience to examine various types of community-based organization that are adaptive to the challenges associated with COVID-19 and continue to provide services to the community residents (30). Finally, the COVID-19 pandemic has also posed an unprecedented demand and a huge burden for healthcare workers (HCWs) including CHWs worldwide, with alarming reports of heightened mental health problems, so protecting and promoting their mental health should receive more attention (31).

## Author contributions

The author confirms being the sole contributor of this work and has approved it for publication.

## References

[1] Harlem G. Descriptive analysis of social determinant factors in urban communities affected by COVID-19. J Public Health. (2020) 42:466–9. 10.1093/pubmed/fdaa078 32530033 PMC7313894

[2] Marshall AT Hackman DA Kan ER Abad S Baker FC Baskin-Sommers A. Location matters: regional variation in association of community burden of COVID-19 with caregiver and youth worry. Health Place. (2022) 77:102885. 10.1016/j.healthplace.2022.102885 35963164 PMC9359938

[3] Ogedegbe G Ravenell J Adhikari S Butler M Cook T Francois F. Assessment of racial/ethnic disparities in hospitalization and mortality in patients with COVID-19 in New York City. JAMA Netw Open. (2020) 3:e2026881. 10.1001/jamanetworkopen.2020.26881 33275153 PMC7718605

[4] Earth Institute. Columbia University Press; New York (2013). One *Million Community Health Workers: Technical Taskforce Report*. Available online at: http://1millionhealthworkers.org/files/2013/01/1mCHW_TechnicalTaskForceReport.pdf (accessed December 1, 2013).

[5] Haines A de Barros EF Berlin A Heymann DL Harris MJ. National UK programme of community health workers for COVID-19 response. Lancet. (2020) 395:10231. 10.1016/S0140-6736(20)30735-2 32220277 PMC7146683

[6] Harris M Haines A. The potential contribution of community health workers to improving health outcomes in UK primary care. J Royal Society of Medicine. (2012) 105:330–5. 10.1258/jrsm.2012.120047 22907550 PMC3423132

[7] United Nations Children's Fund. Minimum Quality Standards and Indicators for Community Engagement. (2020). Available online at: https://www.unicef.org/mena/reports/communityengagement-standards (accessed June 1, 2022).

[8] Pan American Health Organization. COVID-19: Risk Communication and Community Engagement (RCCE). (2020). Available online at: https://www.paho.org/en/file/63164/ (accessed March 2022).

[9] Bedson J Jalloh MF Pedi D Bah S Owen K Oniba A. Community engagement in outbreak response: lessons from the 2014–2016 Ebola outbreak in Sierra Leone. BMJ Global Health. (2020) 5:e002145. 10.1136/bmjgh-2019-002145 32830128 PMC7445350

[10] Yi H Ng ST Farwin A Low AP Chang CM Lim J. Health equity considerations in COVID-19: geospatial network analysis of the COVID-19 outbreak in the migrant population in Singapore. J Travel Med. (2020), 28:taaa159. 10.1093/jtm/taaa159 32894286 PMC7499763

[11] Wai JT Nina G Divya H Dale F. Risk Communication and community engagement during the migrant worker COVID-19 outbreak in Singapore. Sci Commun. (2022) 44:240–51. 10.1177/10755470211061513 35440864 PMC8655121

[12] Wang Q Dai R Zhang T Li J Sheng T Wu B. Supply of basic necessities to vulnerable populations during the COVID-19 pandemic: empirical evidence from Shanghai, China. Front Public Health. (2022) 10:1008180. 10.3389/fpubh.2022.1008180 36388370 PMC9645813

[13] Gilmore B Ndejjo R Tchetchia A de Claro V Mago E Diallo AA. Community engagement for COVID-19 prevention and control: a rapid evidence synthesis. BMJ Glob Health. (2020) 5:e003188. 10.1136/bmjgh-2020-003188 33051285 PMC7554411

[14] Costanza A Amerio A Aguglia A Serafini G Amore M Macchiarulo E. From “the interpersonal theory of suicide”to “the interpersonal trust”: an unexpected and effective resource to mitigate economic crisis related suicide risk in times of Covid-19? Acta Biomed. (2021) 92:e2021417. 34739460 10.23750/abm.v92iS6.12249 PMC8851025

[15] National Association of Community Health Centers. Infographics: National Findings on Health Centers' Response to COVID-19. (2021). Available online at: https://www.nachc.org/research-and-data/research-fact-sheets-and-infographics/infographics-national-findings-on-health-centers-response-to-covid-19/ (accessed December 1, 2021).

[16] Kruse GR Pelton-Cairns L Taveras EM Dargon-Hart S Gundersen DA. Implementing expanded COVID-19 testing in Massachusetts community health centers through community partnerships: protocol for an interrupted time series and stepped wedge study design. Contemp Clin Trials. (2022) 118:106783. 10.1016/j.cct.2022.106783 35533978 PMC9076025

[17] Diaz D Chacko S Sperling A Fleck E Louh I Trepp R. Assessment of digital and community-based outreach interventions to encourage COVID-19 vaccination uptake in an underserved community. JAMA Netw Open. (2022) 5:e2217875. 10.1001/jamanetworkopen.2022.17875 35731520 PMC9218850

[18] Henke RM Johann J Senathirajah M Crandell M Parker L Riley L. Implementation of the patient-centered medical home model in facilities providing comprehensive care to medically underserved populations. J Health Care Poor Underserved. (2016) 27:1638–46. 10.1353/hpu.2016.0152 27818428

[19] Vazquez R Navarrete A Nguyen AT Montiel GIA. Voice to uplift other people: a case study of integrating organizing methods in an FQHC-based COVID-19 vaccine initiative in latinx communities. J Humanist Psychol. (2022) 58:239–61. 10.1177/00221678221125330

[20] Magesh S John D Li WT Li Y Mattingly-App A Jain S. Disparities in COVID-19 outcomes by race, ethnicity, and socioeconomic status: a systematic-review and meta-analysis. JAMA Netw Open. (2021) 4:e2134147. 10.1001/jamanetworkopen.2021.34147 34762110 PMC8586903

[21] Reitsma MB Goldhaber-Fiebert JD Salomon JA. Quantifying and benchmarking disparities in COVID-19 vaccination rates by race and ethnicity. JAMA Netw Open. (2021) 4:e2130343. 10.1001/jamanetworkopen.2021.30343 34668949 PMC8529412

[22] Stillman S Tonin M. Communities and testing for COVID-19. Eur J Health Econ. (2022) 23:617–25. 10.1007/s10198-021-01385-y 35169950 PMC9135992

[23] Xu BL Zhu YB. A systematic review and meta-analysis of the factors associating the willingness of Chinese community residents to receive COVID-19 vaccine. Ann Palliat Med. (2022) 11:P.3483–93. 10.21037/apm-22-1099 36464966

[24] Jones BL Phillips F Shanor D VanDiest H Chen Q Currin-McCulloch J. The leading role of social work at the medical school. A coordinated, compassionate response to COVID-19. Soc Work Health Care. (2021) 60:P.49–61. 10.1080/00981389.2021.1885567 33557718

[25] Okonkwo NE Aguwa UT Jang M Barré IA Page KR Sullivan PS. COVID-19 and the US response: accelerating health inequities. BMJ Evid-Based Med. (2021) 26:176–9. 10.1136/bmjebm-2020-111426 32493833 PMC7299650

[26] Cerami C Canevelli M Santi GC Galandra C Dodich A Cappa SF. Identifying frail populations for disease risk prediction and intervention planning in the Covid-19 era: a focus on social isolation and vulnerability. Front Psychiatry. (2021) 12:626682. 10.3389/fpsyt.2021.626682 34489745 PMC8417585

[27] Abu Assab M Jaber D Haneen B Abu Assab H Al-Atram H. The COVID-19 pandemic and patient safety culture: a cross-sectional study among community pharmacies in jordan. Healthcare. (2022) 10:1434. 10.3390/healthcare10081434 36011091 PMC9408387

[28] Bektay MY Okuyan B Sancar M Izzettin FV. Insight into Turkish community pharmacists' services in the first wave of the COVID-19 pandemic. Disaster Med Public Health Prep. (2021) 1–2. 10.1017/dmp.2021.185 34099093 PMC8314052

[29] He ZF Xiang Y Qi CP Liu XH Li SS. The emergence and impact of community resilience initiatives during the COVID-19 pandemic: an exploration study from a Chinese urban community. Asia Pac J Soc Work Dev. (2022) 32:262–77. 10.1080/02185385.2022.2136235

[30] Altheimer I Duda-Banwar J Schreck C. The impact of Covid-19 on community-based violence interventions. Am J Crim Just. (2020) 45:810–9. 10.1007/s12103-020-09547-z 32837165 PMC7305054

[31] Halms T Strasser M Kunz M Hasan A. How to reduce mental health burden in health care workers during COVID-19?–a scoping review of guideline recommendations. Front Psychiatry. (2022) 12:770193. 10.3389/fpsyt.2021.770193 35126194 PMC8811254

